# Supplementary material for: A cryogenic on-chip microwave pulse generator for large-scale superconducting quantum computing
Source: Nat Commun. 2024 Jul 16;15:5958. doi: 10.1038/s41467-024-50333-w (PMC11251047; doi:10.1038/s41467-024-50333-w)
Supplement: Supplementary file 1 — Supplementary Information [file 41467_2024_50333_MOESM1_ESM.pdf]

Supplementary Information for "A cryogenic on-chip microwave pulse generator for large-scale superconducting quantum computing"

SUPPLEMENTARY NOTE 1: THEORETICAL MODEL

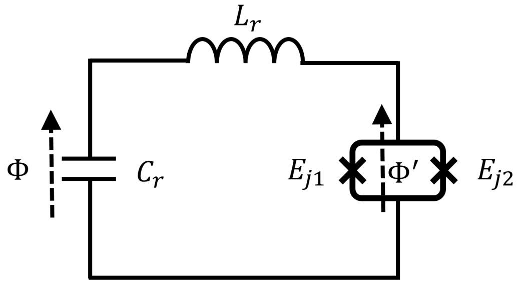

Supplementary Fig. 1. **Schematics of lumped element equivalent circuit.**

In this note, we present a theoretical model to describe the microwave photon generation process. The lumped element equivalent of the circuit is shown in Supplementary Fig. 1. The Lagrangian of the system can be described as,

$$L(\Phi, \Phi') = -\frac{(\Phi - \Phi')^2}{2L_r} + \frac{1}{2}C_r\dot{\Phi}^2 + E_{j1}\cos\frac{2\pi}{\Phi_0}\left(\Phi' - \frac{1}{2}\Phi_{ext}\right) + E_{j2}\cos\frac{2\pi}{\Phi_0}\left(\Phi' + \frac{1}{2}\Phi_{ext}\right), \tag{1}$$

where C_r and L_r are the capacitance and the inductance of the CPW resonator, E_{j1} and E_{j2} are the Josephson energy of the two junctions of the SQUID, Φ, Φ' are the phase differences between the two nodes of the capacitance and the SQUID, Φ_{ext} represents the magnetic flux applied externally through the SQUID loop. In this Lagrangian, the first and second terms are the energy stored in the inductance and capacitance of the CPW resonator, and the third and fourth terms correspond to the energy stored in the two junctions of the SQUID, respectively. According to the Lagrange equation, we can obtain the constraint equation between Φ and Φ' in the dynamical evolution as

$$\frac{\Phi - \Phi'}{L_r} - \frac{2\pi}{\Phi_0}E_{j1}\sin\frac{2\pi}{\Phi_0}\left(\Phi' - \frac{\Phi_{ext}}{2}\right) - \frac{2\pi}{\Phi_0}E_{j2}\sin\frac{2\pi}{\Phi_0}\left(\Phi' + \frac{\Phi_{ext}}{2}\right) = 0, \tag{2}$$

We introduce a parameter δ to characterize the asymmetry of the two junctions in the SQUID. With this, the Josephson energy of the two junctions can be denoted as $E_{j1} = E_0(1-\delta)/2$ and $E_{j2} = E_0(1+\delta)/2$, where $E_0 = E_{j1} + E_{j2}$ represents the total Josephson energy of the two Josephson. Notably, δ ranges from 0 to 1, which stands for a fully symmetric SQUID or a single Josephson junction, respectively. Thus, Supplementary Eq. 2 can be rewritten into a compact form as

$$\begin{aligned} \frac{\Phi_0}{2\pi}\frac{\Phi - \Phi'}{L_r} &= E_j \sin\left(\frac{2\pi}{\Phi_0}\Phi' + \theta\right) \\ E_j &= E_0 \cos\left(\frac{2\pi}{\Phi_0}\frac{\Phi_{ext}}{2}\right)\sqrt{1 + \delta^2\tan^2\left(\frac{2\pi}{\Phi_0}\frac{\Phi_{ext}}{2}\right)}, \end{aligned} \tag{3}$$

where E_j is the flux-dependent Josephson energy of the SQUID. The phase offset θ induced by the externally applied magnetic flux satisfies $\tan\theta = \delta\tan(\pi\Phi_{ext}/\Phi_0)$. When $2\pi\Phi'/\Phi_0 + \theta \ll 1$, we can take the approximation $\sin(2\pi\Phi'/\Phi_0 + \theta) \approx 2\pi\Phi'/\Phi_0 + \theta$, and obtain the Kirchhoff's Current Law for this circuit,

$$\frac{L_r}{L_j}\left(\Phi' + \frac{\Phi_0}{2\pi}\theta\right) = (\Phi - \Phi'), \tag{4}$$

where the effective inductance of the SQUID can be determined by $L_j = (\Phi_0/2\pi)^2/E_j$. According to Supplementary Eq. 4, we can have the relation between the phase variables Φ and Φ' as

$$\Phi' = \frac{L_j\Phi - L_r\frac{\Phi_0}{2\pi}\theta}{L_r + L_j} \tag{5}$$

$$\Phi' + \frac{\Phi_0}{2\pi}\theta = \frac{L_j}{L_r + L_j}\left(\Phi + \frac{\Phi_0}{2\pi}\theta\right). \tag{6}$$

With this relation, we can simplify Supplementary Eq. 1 and obtain the circuit Lagrangian of only phase variable Φ as:

$$L(\Phi) = -\frac{1}{2\left(L_r + L_j\right)}\left(\Phi + \frac{\Phi_0}{2\pi}\theta\right)^2 + \frac{1}{2}C_r\dot{\Phi}^2. \tag{7}$$

Here we take the approximation $\cos\left(2\pi\Phi'/\Phi_0 + \theta\right) \approx 1 - (2\pi\Phi'/\Phi_0 + \theta)^2/2$. From the Lagrangian of the circuit, we can derive the corresponding Hamiltonian as

$$\hat{H} = \frac{1}{2\left(L_r + L_j\right)}\left(\hat{\Phi} + \frac{\Phi_0}{2\pi}\theta\right)^2 + \frac{\hat{Q}^2}{2C_r}, \tag{8}$$

where $\hat{Q} = C_r\dot{\Phi}$ is the corresponding conjugate variables of the phase $\hat{\Phi}$. With the Hamiltonian, we can define the creation and annihilation operators for the bare resonator mode, and the generalized phase $\hat{\Phi}$ and charge $\hat{Q}$ operators can be expressed in the following form

$$\begin{aligned}\hat{\Phi} &= \sqrt{\frac{\hbar Z_r}{2}}\left(\hat{a}^\dagger + \hat{a}\right)\\ \hat{Q} &= i\sqrt{\frac{\hbar}{2Z_r}}\left(\hat{a}^\dagger - \hat{a}\right),\end{aligned} \tag{9}$$

where $Z_r = \sqrt{L_r/C_c}$ is the characteristic impedance of the CPW resonator. By substituting Supplementary Eq. 9 into Supplementary Eq. 8, the Hamiltonian is expanded to a single-mode quadratic form as

$$\hat{H} = \frac{\hbar\omega_0}{4}\left(\frac{L_r}{L_{tot}} - 1\right)\left(\hat{a}^2 + \hat{a}^{\dagger 2}\right) + \frac{\hbar\omega_0}{2}\left(\frac{L_r}{L_{tot}} + 1\right)\hat{a}^\dagger\hat{a} + \frac{\Phi_0}{2\pi}\sqrt{\frac{\hbar L_r\omega_0}{2}}\frac{\theta}{L_{tot}}\left(\hat{a} + \hat{a}^\dagger\right), \tag{10}$$

where $\omega_0 = \sqrt{1/L_rC_r}$ is the bare frequency of the CPW resonator; $L_{tot} = L_r + L_{Jeff}$ is the total inductance of the SQUID-embedded resonator. Notably, the Hamiltonian is associated with a six-dimensional Lie algebra $\left[\hat{a}^{\dagger 2}/2, \hat{a}^2/2, \hat{a}^\dagger\hat{a} + 1/2, \hat{a}^\dagger, \hat{a}, I\right]$ and can be numerically calculated with the method mentioned in the next note without photon number cutoff. In order to unveil the relation between the operator of the bare resonator mode and the one of SQUID-embedded resonator mode, the Hamiltonian can be diagonalized with the transformation

$$\begin{aligned}\hat{a}_t^\dagger &= \frac{1}{2}\left(\sqrt{\frac{Z_r}{Z_t}} + \sqrt{\frac{Z_t}{Z_r}}\right)\hat{a}^\dagger + \frac{1}{2}\left(\sqrt{\frac{Z_r}{Z_t}} - \sqrt{\frac{Z_t}{Z_r}}\right)\hat{a} + \frac{1}{2}\frac{\Phi_0}{2\pi}\theta\sqrt{\frac{2}{Z_t\hbar}}\\ \hat{a}_t &= \frac{1}{2}\left(\sqrt{\frac{Z_r}{Z_t}} + \sqrt{\frac{Z_t}{Z_r}}\right)\hat{a} + \frac{1}{2}\left(\sqrt{\frac{Z_r}{Z_t}} - \sqrt{\frac{Z_t}{Z_r}}\right)\hat{a}^\dagger + \frac{1}{2}\frac{\Phi_0}{2\pi}\theta\sqrt{\frac{2}{Z_t\hbar}},\end{aligned} \tag{11}$$

where $Z_t = \sqrt{L_{tot}/C_r}$ and $\hat{a}_t(\hat{a}_t^\dagger)$ are the characteristic impedance and annihilation (creation) operator of the SQUID-embedded resonator mode. With this transformation, the Hamiltonian can be rewritten as a frequency-tunable harmonic oscillator form

$$\hat{H} = \hbar\omega\left(\hat{a}_t^\dagger\hat{a}_t + \frac{1}{2}\right), \tag{12}$$

where $\omega = \sqrt{1/L_{tot}C_r}$ is the resonant frequency of the SQUID-embedded resonator mode, which is dependent on the external magnetic flux.

SUPPLEMENTARY NOTE 2: NUMERICAL SIMULATION

With the theoretical model derived above, the dynamical evolution of the microwave emission process can be calculated numerically [1]. According to Supplementary Eq. 10, the time-evolution operator for this single-mode quadratic Hamiltonian can be separated into the following form,

$$\hat{U}(t) = e^{i\varphi(t)} \hat{S}(z(t)) \hat{D}(\alpha(t)) \hat{R}(\xi(t)), \tag{13}$$

where $\hat{S}(z(t)) = \exp\left(z(t)\hat{a}^{\dagger 2}/2 - z(t)^* \hat{a}^2/2\right)$, $\hat{D}(\alpha(t)) = \exp\left(\alpha(t)\hat{a}^\dagger - \alpha(t)^* \hat{a}\right)$, and $\hat{R}(\xi(t)) = \exp\left(i\xi(t)\hat{a}^\dagger\hat{a}\right)$ are the single-mode squeeze, displacement and rotation operators, respectively, of which $z(t) = r\exp(i\psi)$ is the squeeze factor with $r \geq 0$ and $2\pi \geq \psi \geq 0$, $\alpha(t)$ is the complex displacement amplitude and $\xi(t)$ is the real rotation angle. Here, the time-evolution operator is arranged into a product of the single-mode squeeze, displacement, and rotation operator with an overall phase factor $\varphi(t)$. For the convenience of numerical calculation, Supplementary Eq. 13 can be found in a normal-ordered form as

$$U(t) = \exp[A(t)] \exp\left[B(t)\hat{a}^\dagger + C(t)\hat{a}^{\dagger 2}\right] \sum_{n=0}^{\infty} \frac{:\left[D(t)\hat{a}^\dagger\hat{a}\right]^n:}{n!} \exp\left[E(t)\hat{a} + F(t)\hat{a}^2\right] \tag{14}$$

With this expression, the calculation for the time-evolution operator or the time-dependent density matrices of the system can be converted into solving the time-dependent parameters A, B, C, D, E, F, which can be given by the following equations,

$$\begin{aligned}
i\dot{A} &= f^*\left(2C + B^2\right) + g^* B \\
i\dot{B} &= (4Cf^* + v) B + 2Cg^* + g \\
i\dot{C} &= 4f^* C^2 + 2vC + f \\
i\dot{D} &= (4Cf^* + v)(D+1) \\
i\dot{E} &= (D+1)(2f^* B + g^*) \\
i\dot{F} &= f^*(D+1)^2,
\end{aligned} \tag{15}$$

where $v = \omega_0 (L_r/L_{tot} + 1)/2$, $f = \omega_0 (L_r/L_{tot} - 1)/4$, and $g = (\Phi_0\theta/2\pi L_{tot})\sqrt{L_r\omega_0/2\hbar}$ are the parameters determined by the system Hamiltonian Supplementary Eq. 10. According to Supplementary Eq. 13 and Supplementary Eq. 14, the relation between the two sets of time-dependent parameters A, B, C, D, E, F and z, α, ξ can be derived as

$$\begin{aligned}
B &= \operatorname{sech}(r)\,\alpha \\
C &= 1/2 \tanh(r)\, e^{i\psi} \\
D &= \operatorname{sech}(r)\, e^{i\xi} - 1 \\
E &= -\frac{2C^* B + B^*}{D^* + 1} \\
F &= -\frac{C^*(D+1)}{D^* + 1} \\
\varphi &= \operatorname{Im}\left[A + C^*\alpha^2\right],
\end{aligned} \tag{16}$$

From Supplementary Eq. 11, the initial condition of the SQUID-embedded harmonic oscillator can be determined as $z(0) = \ln\sqrt{Z_r/Z_t}, \alpha(0) = -(\Phi_0\theta(0)/2\pi)\sqrt{1/2\hbar Z_r}, \xi(t) = 0$. With Supplementary Eq. 15, the time evolution of the system can be calculated without photon number cutoff. When the time-dependent parameters are determined, the expectation values of the annihilation operator and photon number operator of the SQUID-embedded resonator mode can be calculated as

$$\langle \hat{a}_t \rangle = \cosh(r')\alpha + \sinh(r')e^{i\psi'}\alpha^* + \sqrt{\frac{1}{2\hbar Z_r}} \frac{\Phi_0}{2\pi}\theta \tag{17}$$

$$\left\langle \hat{a}_t^\dagger \hat{a}_t \right\rangle = \|\langle \hat{a}_t \rangle\|^2 + \sinh^2(r'), \tag{18}$$

where $r' \exp(i\psi') = z(t) - \ln\sqrt{Z_r/Z_t}$ and α are the calculated time-dependent parameters. Note that the above-discussed excitation is on the internal mode of the SQUID-embedded resonator $\hat{a}_t$. For the microwave emission output from the resonator, the internal loss and out-coupling rates should be taken into account. Since the decay time constant of the resonator is much larger than the timescale of the photon generation process, the photon loss during the dynamic evolution can be neglected.

In Supplementary Fig. 2, we show the numerical simulation results of the microwave photon generation process with a set of fixed sample parameters similar to that used in the experiment. From Supplementary Fig. 2**a**, one can find that the photon generation occurs when the flux step goes across the $\Phi_0/2$ point. The time traces of the expectation value $\| \langle \hat{a}' \rangle \|^2$ and $\langle \hat{a}'^\dagger \hat{a}' \rangle$ are almost coincident with each other, which indicates that the coherent component is dominant in the generated photon state. In Supplementary Fig. 2**b**, the output power of the microwave emission is calculated with a varied initial flux of the flux step. The microwave emission power shows a sharp increase when initial flux goes beyond $\Phi_0/2$, which is consistent with the experimental results shown in Fig. 2**d** of the main text. The phase of the microwave emission is extracted as shown in Supplementary Fig. 2**c**, showing a continuous evolution ranging several periods with a varied initial flux, which aligns with the experimental results shown in Fig. 2**e** of the main text.

We further analyze the emission power dependence on the slope of the flux step and the junction asymmetry of the SQUID, as shown in Supplementary Fig. 3. The simulation results predict that a faster flux change rate or a smaller junction asymmetry is helpful in improving the microwave emission intensity.

We note that the current toy model serves as a qualitative and semi-quantitative explanation of the microwave photon generation process. When the approximation condition $2\pi\Phi'/\Phi_0 + \theta \ll 1$ is no longer satisfied (the dashed line region in Supplementary Fig. 3**b** when asymmetry δ is smaller than about 0.026), the numerical simulation reliability reduces. Nevertheless, the model is sufficient to provide an intuitive explanation of the microwave pulse generation.

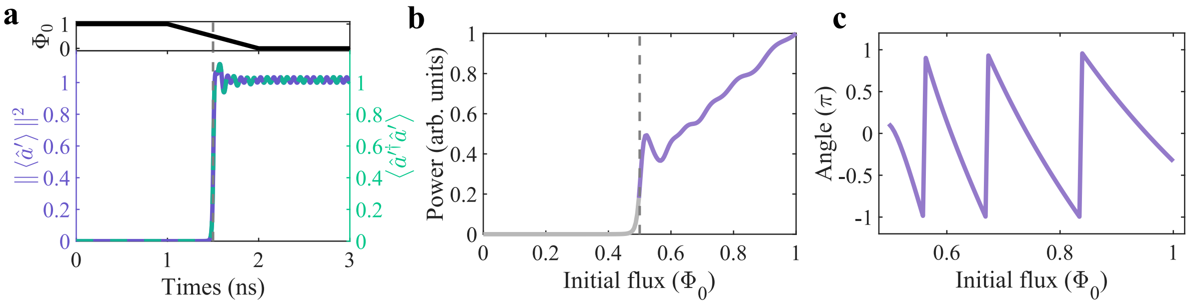

Supplementary Fig. 2. **Microwave photon generation predicted by the theoretical model.** **a**, Theoretically predicted time evolution of the square of the absolute value of annihilation operator $\| \langle \hat{a}' \rangle \|^2$ (purple solid line) and the photon number operator $\langle \hat{a}'^\dagger \hat{a}' \rangle$ (cyan dashed line) of the resonator mode, when a magnetic flux step (the top panel) is applied as drive, which changes from the initial flux Φ_0 to the end flux 0 linearly in 1 ns. Both the expectation values are normalized to the final photon number. The timing when the flux drive goes across the $\Phi_0/2$ point is marked with the grey dashed line. **b** and **c**, Theoretically predicted normalized power and phase of the microwave output driven with a varied initial flux. The $\Phi_0/2$ point is indicated by the grey dashed line. The numerical calculation is conducted with SQUID Josephson energy $E_0 = 2.7$ THz, junction asymmetry $\delta = 0.0526$, and resonator bare frequency $\omega_0 = 7$ GHz.

SUPPLEMENTARY NOTE 3: CALIBRATION OF THE OUTPUT POWER

The output power of the signal source can be determined with the knowledge of the gain of the amplifier chain shown in Extended Data Fig. 1 of the main text. To this aim, we use measurement-induced dephasing of a superconducting qubit to bridge the circuit photon number and the homodyne signal [2]. Considering the superconducting transmon qubit and the resonator dispersively coupled with the qubit with the circuit quantum electrodynamics model, if we populate the resonator with a certain photon flux, the photons in the resonator would introduce a dephasing term to the qubit state via AC stark effect. Accordingly, we could determine the resonator photon number by measuring the qubit dephasing with a Ramsey method.

When a continuous coherent drive with photon flux $\dot{n}_d$ at frequency ω_d is sent to the resonator, the additional qubit

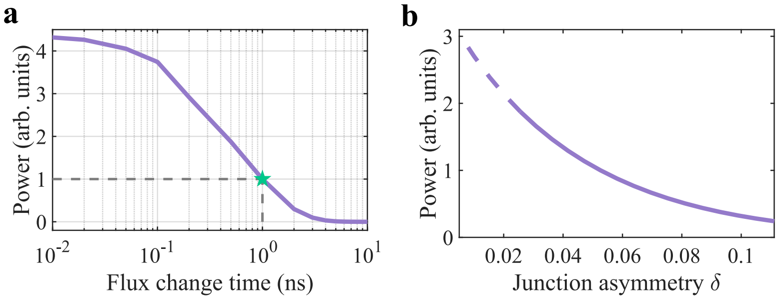

Supplementary Fig. 3. **Predicted emission power dependence on the drive and sample parameters.** **a**, Theoretically predicted power of the microwave emission with a varied flux change time, when the initial flux and the end flux are fixed at Φ_0 and 0, respectively, and the junction asymmetry takes $\delta = 0.0526$. The power is normalized with the intensity when the flux change time is 1 ns (cyan star), which corresponds to the upper limit of our experimental setup. **b**, Theoretically predicted power of the microwave emission with varied junction asymmetry δ, when the initial flux, end flux, and the flux change time are fixed at Φ_0, 0, and 1 ns, respectively. The power is normalized with the case when $\delta = 0.0526$. The region $\delta < 0.0256$ is marked with a dashed line due to the possible distortion induced by the invalid approximation. The numerical calculation is conducted under the sample parameter SQUID Josephson energy $E_0 = 2.7$ THz and resonator bare frequency $\omega_0 = 7$ GHz.

dephasing rate Γ_m would be [2]

$$\begin{aligned} \Gamma_m &= \frac{\kappa_{tot}\chi^2}{\kappa_{tot}^2/4 + \chi^2 + \Delta_d^2}(\bar{n}_+ + \bar{n}_-) \\ \bar{n}_\pm &= \frac{\kappa_r \dot{n}_d}{\kappa_{tot}^2/4 + (\Delta_d \pm \chi)^2}, \end{aligned} \tag{19}$$

where $\bar{n}_\pm$ is the average photon number in the resonator when the qubit is in $|g\rangle$ or $|e\rangle$. $\Delta_d = \omega_d - \omega_c$ is the detuning between the coherent driving and resonator frequency, κ_c is the decay rate of the input port for the photon flux, and κ_{tot} is the total decay rate of the resonator. The input photon flux $\dot{n}_d$ can be determined with Supplementary Eq. 19 by measuring the qubit dephasing rate at varied input signal strength. The photon flux reflected by the resonator $\dot{n}_{out}$ can be determined as

$$\dot{n}_{out} = \left| \frac{i\kappa_c}{(\Delta_d - \chi) + i\frac{\kappa_{tot}}{2}} - 1 \right|^2 \dot{n}_d \tag{20}$$

The photon flux $\dot{n}_{out}$ is sent to the amplifier chain and analyzed by the homodyne setup as a voltage signal. Therefore, we can calibrate the homodyne measurement result to a certain photon flux or photon number, which is essentially the effective gain of the detection setup.

In the experiment, we use a commercial signal source and AWG to prepare a square pulse that fits the bandwidth of the resonator to calibrate the effective gain of the homodyne setup. With this, we can determine both the energy (total photon number) contained in the microwave pulses and the power of the CW output from the cryogenic signal source.

SUPPLEMENTARY NOTE 4: USING TWISTED-PAIR WIRES TO DELIVER THE CONTROL SIGNAL

In most of the experiments, we use coaxial cables to deliver the control signal of the cryogenic source from room temperature to cryogenic temperature to prepare microwave emissions. Since the magnetic flux step used to generate the microwave emission is akin to a digital signal, it would be beneficial if twisted-pair wires could be used for the control signal delivery, considering that the passive heat load of the twisted-pair wires is much lower than that of a coaxial cable [3]. In the experiment, we use twisted pairs of superconducting (NbTi) wires to connect the room-temperature pulse generator and the cryogenic microwave signal source, as illustrated in Extended Data Fig. 1 in the main text. It is reported that the passive heat load of such wires is six orders smaller than that for the commonly used stainless steel coaxial cable, thus allowing larger-scale integration.

We first characterize the distortion of a signal edge delivered through the cold twisted pairs in the fridge. The well-heatsinked twisted pairs of wires are used as input channels and directly connected to a coaxial cable line as the output channel. A square pulse with a time resolution of 1 ns is prepared with the AWG as input and the output, as shown in Supplementary Fig. 5, is recorded by an oscilloscope with a sampling rate of 10 GHz. Even though the

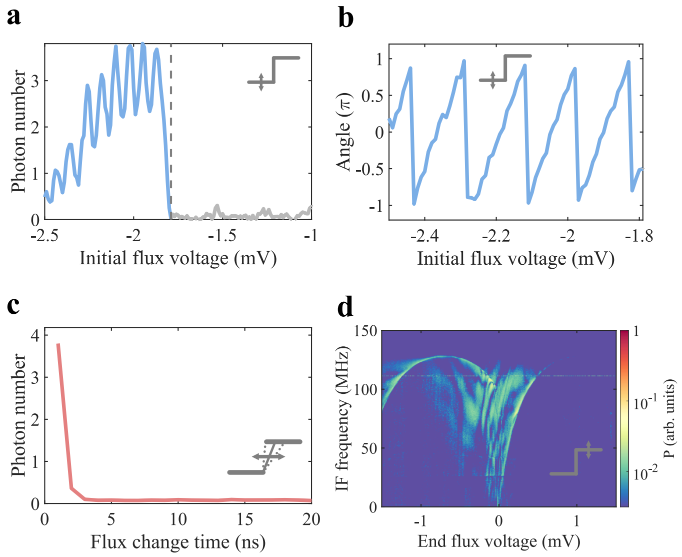

Supplementary Fig. 4. **Microwave emission driven through twisted-pair wires.**

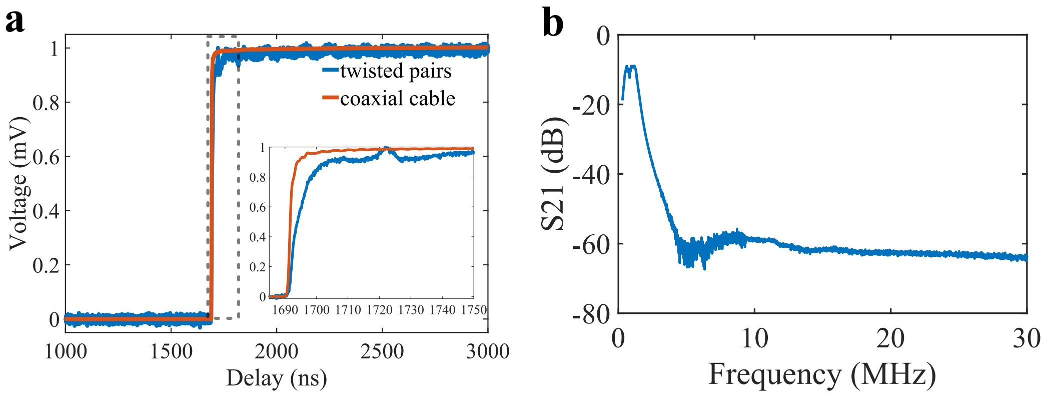

Supplementary Fig. 5. **Influence of the limited bandwidth of the twisted-pair wires a**, Distortions of a signal edge for coaxial cable and twisted-pair wires. **b**, The transmission measurement shows a typical bandwidth of the twisted pairs of smaller than 5 MHz. Even though the twisted-pair wires have limited bandwidth, it is still capable of delivering the flux edge used to drive the microwave signal source from room temperature to cryogenic temperature. The limited bandwidth of the twisted-pair wires distorts the flux edge from its ideal shape more than that of the coaxial cable, which explains the decreased emission energy when the signal source is driven through the twisted-pair wires.

signal edge is distorted from its ideal shape, its general shape is retained through the twisted pairs, manifesting the possibility of using the twisted pairs for the delivery of the stepped signal used to drive the cryogenic microwave.

Using the twisted pairs as flux input channels, Supplementary Fig. 4**a** and **b** show the recorded microwave emission using varied initial values of the flux step as drive. It can be seen that coherent microwave signals with well-controlled initial phases can be generated once the magnetic flux exceeds the thresholds. In Supplementary Fig. 4**c**, the energy of the emitted microwave pulse is reduced with a smaller slop of the flux edge. Meanwhile, the frequency of the microwave emission can be tuned using varied end values of the flux step, as shown in Supplementary Fig. 4**d**. All those results show similar phenomena as that illustrated in Fig. 2 of the main text, verifying that the twisted-pair wires can be used to deliver the stepped magnetic flux to drive the cryogenic microwave source.

We note that in Supplementary Fig. 4**d**, the frequency of the microwave output driven through the twisted-pair wires is not as clean as that with the coaxial cable. This is attributed to the impedance mismatch between the AWG output and the twisted-pair wires, leading to an unstable output of the AWG, which is also evidenced by the unstable and broadened spectra of the signal source. In the meantime, the energy of the microwave pulse generated with the twisted-pair wires is smaller than that with the coaxial cable (typically about 0.07 times that of the coaxial cables). Such a result is not surprising considering that the twisted-pair wires would distort and reduce the edge slope of the signal step (Supplementary Fig. 5**a**), thus reducing the emission energy. Such a distortion originates from the limited

bandwidth of the twisted pairs, as shown in Supplementary Fig. 5**b**. It has been reported that by optimizing the twist pitch length of the pair of wires, the bandwidth can be improved to even several gigahertz [4], and thus a much larger edge slope of the signal step seen by the pulse generator.

SUPPLEMENTARY NOTE 5: EXTENDED DATA

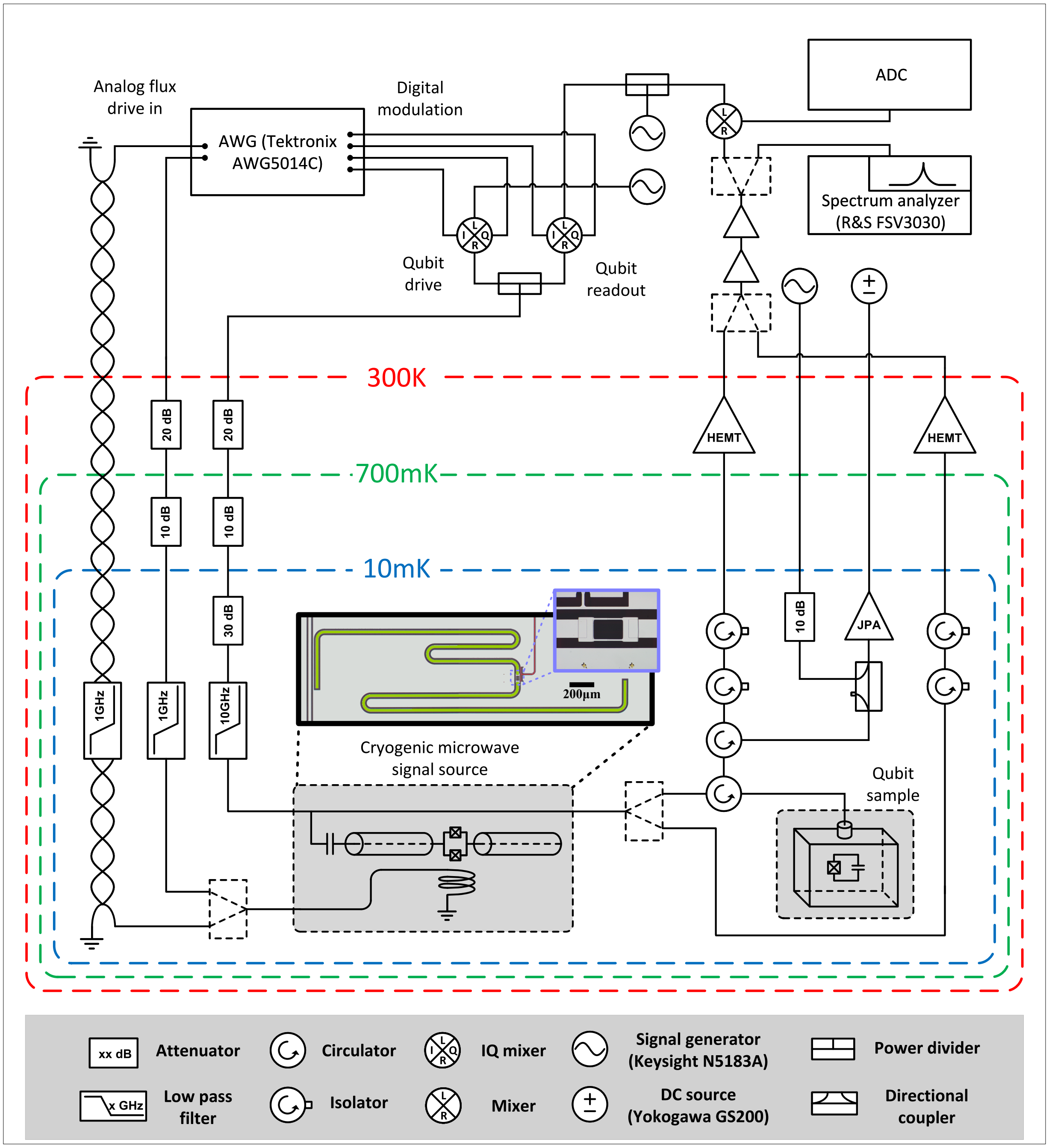

Supplementary Fig. 6. **Schematic diagram of the experimental setup.** The colored dashed boxes depict the different temperature stages of the dilution refrigerator. The shadowed boxes are schematics of the cryogenic microwave signal source and the qubit sample for the readout experiment, respectively. The inset shows optical microscope images of the signal source that consists of a SQUID-embedded CPW resonator (green). The flux step or overshoot used to drive the signal source is generated with an AWG, and delivered through either coaxial cables or superconducting twisted-pair wires to the signal source located at the 10 mK region. The dashed boxes enclosed area designate different circuit connections depending on the specific measurements. For the basic characterization of the cryogenic signal source, the output of the signal source is amplified by the amplifier chain containing a HEMT amplifier and two room-temperature amplifiers and sent to the homemade homodyne setup or the spectrum analyzer. For the qubit readout experiment, the output of the signal source is sent to the transmon qubit sample. The reflected signal from the qubit readout resonator is first sent to the amplifier chain started with a Josephson parametric amplifier and analyzed by the homodyne setup.

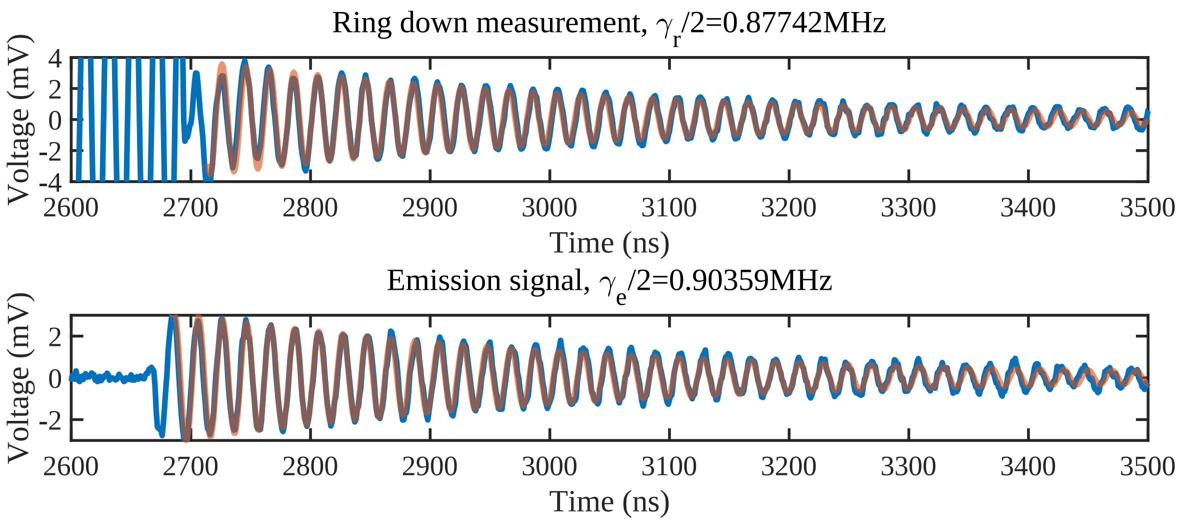

Supplementary Fig. 7. **Decay time constant for the emission pulse.** The upper panel shows a ring-down measurement for the SQUID-embedded resonator. The lower panel shows a time series of the emitted microwave pulse. By fitting with an exponential enveloped sinusoidal function, the two measurement results show similar decay time constants, manifesting that the decay time constant for the emission pulse is determined by the linewidth of the resonator. The slight difference in the time constants between the ring-down measurement and the microwave emission is due to the nonlinearity of the SQUID-embedded resonator, for which the photon population in the resonator would influence its resonance frequency and linewidth.

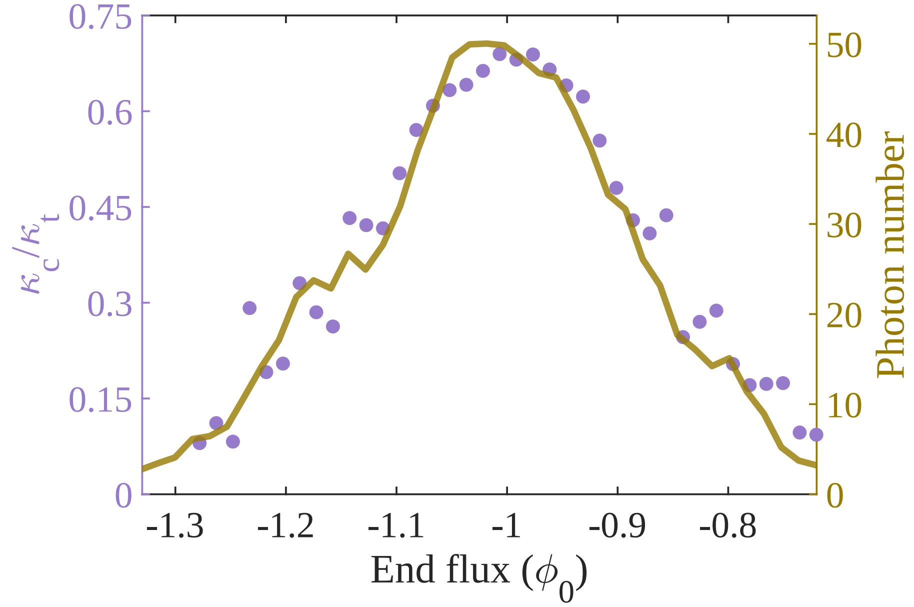

Supplementary Fig. 8. **The dependence of output photon number on the internal loss rate of the resonator.** The black scattered plots show κ_c/κ_t as a function of resonance frequency. κ_c and κ_t are the out-coupling rate and the linewidth of the resonator, respectively, which are extracted by fitting the reflection spectra in the main text Fig. 2**a**. κ_c/κ_t estimates the proportion of the out-coupled emission energy, which shows similar dependence on the (end) magnetic flux as that for the measured output photon number (solid line). When using a flux step to drive the signal source, the frequency of the microwave emission can be tuned by the end flux, accompanied by varied output photon number, as seen in the main text Fig. 2**g**. The change in output photon number can be explained by the increased internal loss rate κ_i when the frequency of the SQUID-embedded resonator is tuned away from its sweet spot. An increased κ_i would enlarge the resonator linewidth $\kappa_t = \kappa_c + \kappa_i$, and thus decrease the proportion of the out-coupled energy κ_c/κ_t, which nicely reproduces the change of the emitted photon number.

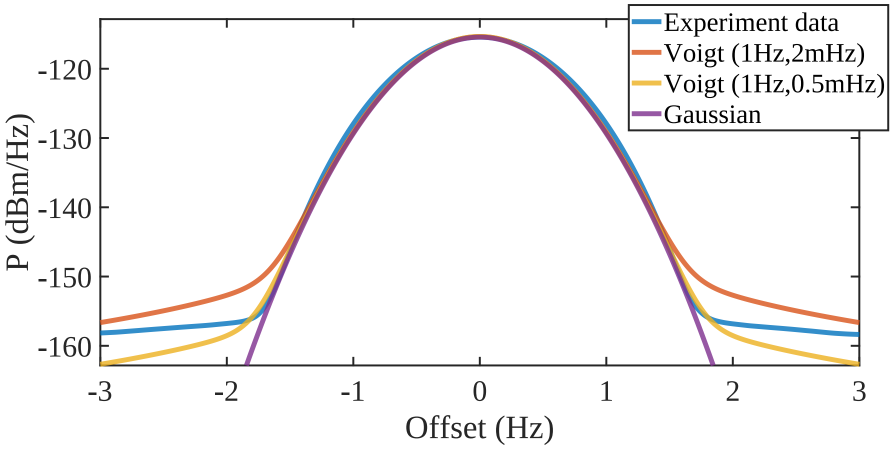

Supplementary Fig. 9. **Linewidth estimation of the CW microwave emission.** The power spectrum density of the CW microwave output around the principal maximum (blue) was measured with a spectrum analyzer. The CW output is prepared by using a train of δ function-like magnetic flux pulses to drive the signal source. The minimum resolution bandwidth of 1 Hz employed in the experiment sets a bottleneck for the signal linewidth measurement, characterized by the Gaussian function with a full width at half maximum (FWHM) of 0.93 Hz (purple). Two additional Voigt fits corresponding to the convolutions of the Gaussian component with 1 Hz FWHM and the Lorentzian components with 2 mHz (orange) and 0.5 mHz (yellow) FWHMs are shown to bound the linewidth of the CW output.

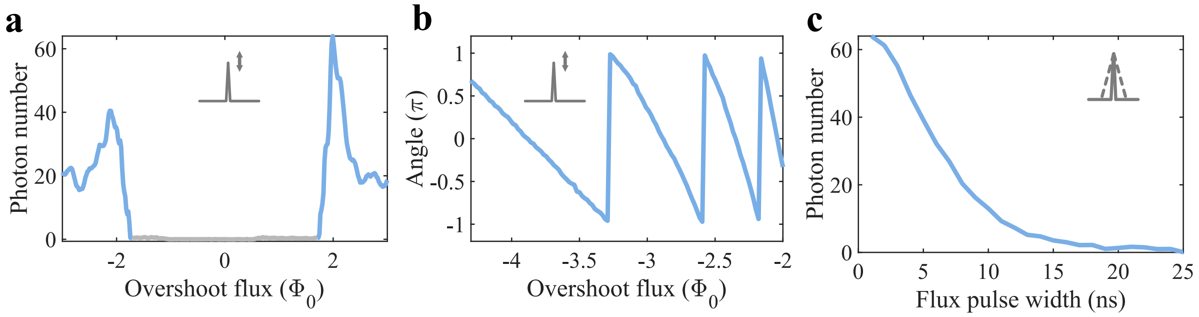

Supplementary Fig. 10. **Microwave emission driven with magnetic flux pulse.** When the cryogenic signal source is driven by a δ-function like magnetic flux pulse, microwave pulses can be generated once the flux overshoots a threshold. **a** and **b**, The photon number and phase of the microwave emission can be continuously tuned by varying the overshoot flux. We note that the threshold here is not the odd multiples of the half flux quantum, compared with the case of using flux edge as drive. This is due to the non-ideal impulse response of the flux pulse generator and also the flux line cabling, which distorts the flux overshoot seen by the cryogenic signal source. **c**, For a fixed flux overshoot, the emission photon number can be continuously tuned with a varied width of the flux pulse. Here the flux pulse is generated with a voltage overshoot outputted from an AWG with a 1 GHz sampling rate, which is delivered through a coaxial cable to the flux line of the signal source. The insets illustrate the applied magnetic flux overshoots.

Supplementary Table I. Sample parameters for the qubit readout experiments

bare frequency of readout resonator, $\omega_c/2\pi$(GHz)	6.5335
internal loss rate of readout resonator, $\kappa_i/2\pi$(MHz)	0.21
out-coupling rate of readout resonator, $\kappa_c/2\pi$(MHz)	2.09
qubit frequency, $\omega_q/2\pi$(GHz)	4.6028
qubit anharmonicity, $E_c/2\pi$(MHz)	343.57
dispersive coupling rate, $\chi_{ge}/2\pi$(MHz)	-1.6
qubit energy relaxation time of $\|e\rangle$, $T_1^{ge}(\mu s)$	43
qubit dephasing time of $\|e\rangle$, $T_2^{ge}(\mu s)$	2

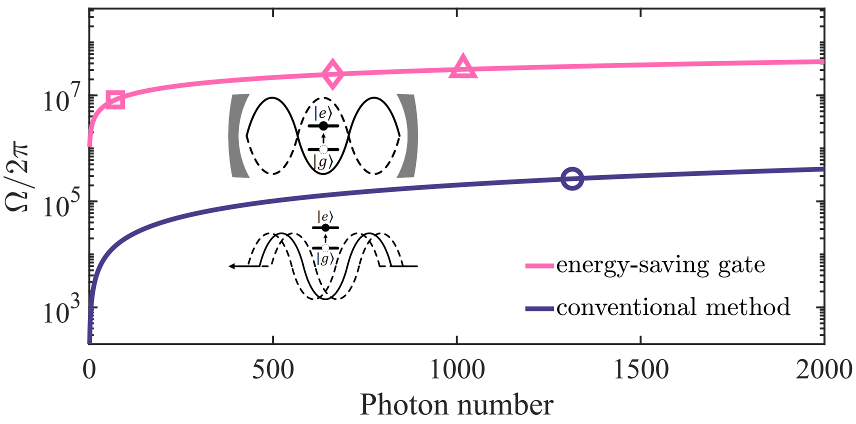

Supplementary Fig. 11. **Qubit drive estimation with the cryogenic microwave source.** The single qubit Rabi rate is theoretically estimated as a function of the emission photon number with the conventional method (purple line) or the energy-saving gate approach (pink line). In the conventional approach, the output of the signal source is connected to the qubit through an open waveguide as the drive line, where the coupling rate between the qubit and the drive line is assumed to be $\Gamma_{ext} = 1000$ Hz for theoretical estimation. In this case, the Rabi rate achieved with the maximum emission output power using coaxial cables is on the order of 0.26 MHz marked by the purple circle. For the energy-saving gate approach, the resonator of the signal source is directly connected to the qubit with a coupling capacitance the same as the conventional method [5, 6]. Thus, the single-qubit operation is realized by resonant Rabi oscillation between the qubit and resonator. The pink triangle and square indicate the Rabi rates 30.8 MHz and 8.23 MHz corresponding to the maximum photon number experimentally generated in the resonator 1017 and 71 when the flux drive signal of the signal source is delivered through the coaxial cables or twisted-pair wires. By optimizing the twist pitch length of the pair of wires and thus their bandwidth [4], the maximum achieved photon number is estimated to be 662 with the Rabi rate 24.9 MHz shown with the pink diamond. The schematic diagrams of the two approaches are shown below the corresponding lines, respectively.

SUPPLEMENTARY REFERENCES

[1] X. Ma and W. Rhodes, Multimode squeeze operators and squeezed states, Phys. Rev. A **41**, 4625 (1990).

[2] S. Kono, K. Koshino, Y. Tabuchi, A. Noguchi, and Y. Nakamura, Quantum non-demolition detection of an itinerant microwave photon, Nature Physics **14**, 546 (2018).

[3] S. Krinner, S. Storz, P. Kurpiers, P. Magnard, J. Heinsoo, R. Keller, J. Lütolf, C. Eichler, and A. Wallraff, Engineering cryogenic setups for 100-qubit scale superconducting circuit systems, EPJ Quantum Technology **6** (2019).

[4] E. Dinc, S. Bukhari, A. Al Rawi, and E. Acedo, Investigating the upper bound of high-frequency electromagnetic waves on unshielded twisted copper pairs, Nature Communications **13** (2022).

[5] K. Igeta, N. Imoto, and M. Koashi, Fundamental limit to qubit control with coherent field, Phys. Rev. A **87**, 022321 (2013).

[6] J. Ikonen, J. Salmilehto, and M. Möttönen, Energy-efficient quantum computing, npj Quantum Information **3** (2017).
